# A Combination of Zinc and Arginine Disrupt the Mechanical Integrity of Dental Biofilms

**DOI:** 10.1128/spectrum.03351-22

**Published:** 2022-12-06

**Authors:** Erin S. Gloag, Yalda Khosravi, James G. Masters, Daniel J. Wozniak, Carlo Amorin Daep, Paul Stoodley

**Affiliations:** a Department of Microbial Infection and Immunity, The Ohio State University, Columbus, Ohio, USA; b Colgate-Palmolive Technology Center, Piscataway, New Jersey, USA; c Department of Microbiology, The Ohio State University, Columbus, Ohio, USA; d Department of Orthopaedics, The Ohio State University, Columbus, Ohio, USA; e National Biofilm Innovation Centre (NBIC), University of Southampton, Southampton, United Kingdom; f National Centre for Advanced Tribology at Southampton (nCATS), Mechanical Engineering, University of Southampton, Southampton, United Kingdom; Griffith University

**Keywords:** biofilm removal, biophysical properties, dentifrice, mechanics, oral biofilm, viscoelasticity

## Abstract

Mechanical cleaning remains the standard of care for maintaining oral hygiene. However, mechanical cleaning is often augmented with active therapeutics that further promote oral health. A dentifrice, consisting of the “Dual Zinc plus Arginine” (DZA) technology, was found to be effective at controlling bacteria using *in vitro* laboratory studies, translating to clinical efficacy to deliver plaque and gingivitis reduction benefits. Here, we used biophysical analyses and confocal laser scanning microscopy to understand how a DZA dentifrice impacted the mechanical properties of dental plaque biofilms and determine if changes to biofilm rheology enhanced the removal of dental plaque. Using both uniaxial mechanical indentation and an adapted rotating-disc rheometry assay, it was found that DZA treatment compromised biofilm mechanical integrity, resulting in the biofilm being more susceptible to removal by shear forces compared to treatment with either arginine or zinc alone. Confocal laser scanning microscopy revealed that DZA treatment reduced the amount of extracellular polymeric slime within the biofilm, likely accounting for the reduced mechanical properties. We propose a model where arginine facilitates the entry of zinc into the biofilm, resulting in additive effects of the two activities toward dental plaque biofilms. Together, our results support the use of a dentifrice containing Dual Zinc plus Arginine as part of daily oral hygiene regimens.

**IMPORTANCE** Mechanical removal of dental plaque is augmented with therapeutic compounds to promote oral health. A dentifrice containing the ingredients zinc and arginine has shown efficacy at reducing dental plaque both *in vitro* and *in vivo*. However, how these active compounds interact together to facilitate dental plaque removal is unclear. Here, we used a combination of biophysical analyses and microscopy to demonstrate that combined treatment with zinc and arginine targets the matrix of dental plaque biofilms, which destabilized the mechanical integrity of these microbial communities, making them more susceptible to removal by shear forces.

## INTRODUCTION

Routine daily oral hygiene is an effective method for preventing oral disease complemented by regular dentist visits. However, the benefits of mechanical cleaning alone by brushing, floss, picks, or water jets can be improved by the addition of antimicrobial agents to daily dentifrices, for efficient dental plaque control. It has previously been shown that a combination of amino acids and salts of heavy metals, such as zinc and arginine, are effective at dental plaque removal (1–5).

Exogenous arginine has been shown to promote healthy plaque homeostasis, by maintaining a neutral pH in the dental plaque biofilm (6–8), promoting the growth of bacteria associated with a healthy dental plaque, and preventing the out-growth of cariogenic organisms (9, 10). Furthermore, exogenous arginine can inhibit microbial coaggregation (11–13) and disrupt dental plaque biofilms (14–16). In support of this, we previously demonstrated that arginine-treated Streptococcus gordonii biofilms detached from surfaces at lower external shear forces compared to untreated biofilms (17). This indicated that arginine treatment weakened the mechanical integrity of the biofilm, making it more susceptible to removal by mechanical shear forces.

Exogenous zinc is bacteriostatic, by inhibiting bacterial metabolism (18–20). Furthermore, zinc inhibition of metabolic enzymes also reduces both acid and alkali production by cariogenic organisms, such as Streptococcus mutans and oral streptococci, including Streptococcus rattus and Streptococcus salivarius, respectively (19, 21). Consistent with this, exogenous zinc can maintain a neutral pH of dental plaque both *in vitro* and *in vivo* (22, 23). Exogenous zinc has also been proposed to reduce microbial colonization and plaque development by altering the bacterial cell surface, by either binding to surface adhesins, or reducing the net negative charge of the cell (24). Finally, exogenous zinc can also potentiate the virulence of periodontal and gingivitis organisms, including Porphyromonas gingivalis and Fusobacterium nucleatum (18, 24, 25). However, the antimicrobial activity of zinc was reduced in biofilms compared to planktonic cultures, which was hypothesized to be due to the high cell density reducing zinc penetration into the biofilm (19, 23), explaining why the bacteriostatic action of zinc was most pronounced in the outer layer of dental plaque biofilms (26). Despite this, exogenous zinc was able to significantly reduce dental plaque formation both *in vitro* and *in vivo*, whereas an increased inhibition was observed in volunteers with high plaque levels compared to those with low plaque levels (26–28).

Recent efforts have focused on the development of dentifrices with a combination of compounds that synergistically reduce dental plaque and maintain healthy homeostasis. In line with this focus, a Dual Zinc plus Arginine (DZA) dentifrice consisting of 0.96% zinc ions as a combination of zinc oxide, zinc citrate, and 1.5% l-arginine was developed (5). These two forms of zinc were selected to modulate the delivery of zinc within the oral cavity. Zinc citrate is water soluble and considered an immediate source of zinc ions, whereas zinc oxide is insoluble in water and considered a slow-release source of zinc ions. l-arginine was found to increase the deposition of zinc on both oral surface mimetics and dental plaque biofilms (16). In line with this, dental plaque biofilms treated with DZA had both reduced metabolic activity, oxygen consumption, and viability, compared to either untreated biofilms or biofilms treated with Dual Zinc (0.96% zinc ions; zinc oxide and zinc citrate) (16). Furthermore, trials in which subjects used a DZA toothpaste as part of their daily oral hygiene regime showed reduced dental plaque and markers of oral disease compared to subjects that used a sodium fluoride control toothpaste (29, 30). Despite these observations, it is still unclear how DZA is impacting the structure and mechanical properties of dental plaque biofilms. Here, we used confocal laser scanning microscopy, uniaxial mechanical indentation, and a novel rotating-disc rheometry assay to address this question.

## RESULTS

### Streptococcus gordonii biofilms treated with Dual Zinc plus Arginine were more susceptible to mechanical removal.

An understanding of biofilm mechanics is important to the development and application of therapeutics because these properties often define how a biofilm responds to chemical and mechanical forms of eradication. Furthermore, changing the biofilm mechanics is associated with improved penetration and, therefore, enhanced action of therapeutic compounds (31, 32). We, therefore, sought to understand how the DZA technology altered the mechanics of biofilms relevant to oral health. We recently adapted a novel rotating-disc rheology assay to analyze and quantify the detachment of arginine-treated Streptococcus gordonii biofilms (17). S. gordonii is a natural commensal of saliva-plaque biofilms and supportive of early colonizers of dental plaque (6, 33–35). We used this assay to determine how treatment with DZA impacted S. gordonii biofilm mechanics and removal when exposed to shear stress.

**FIG 1:**
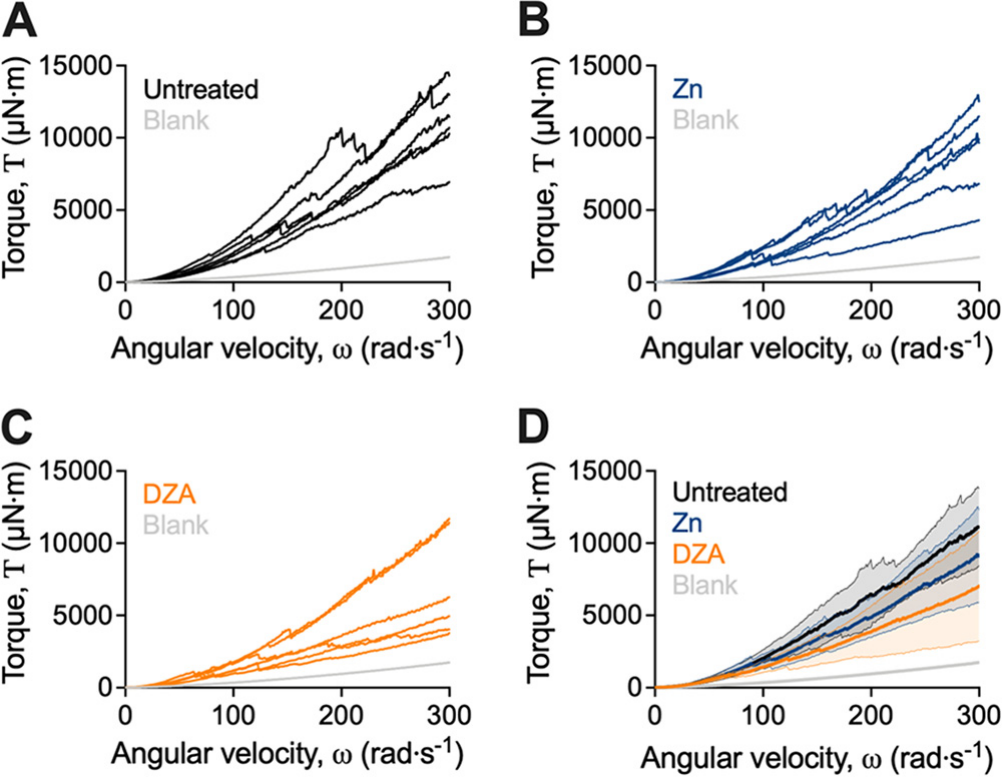
S. gordonii biofilms treated with Dual Zinc plus Arginine display reduced torque when analyzed by adapted rotating-disc rheology. S. gordonii biofilms were grown for 5 days and were treated with either (A) PBS (untreated), (B) 0.96% zinc (Zn), or (C) DZA for 2 min. Biofilms were analyzed by adapted rotating-disc rheology. Data are presented as torque–angular velocity curves of individual replicate biofilms, separated per treatment group for clarity. (D) Data from (A to C) presented as the mean ± 95% confidence interval of combined replicates for direct comparison across treatments. Three biological replicates were performed each with duplicate biofilms.

S. gordonii biofilms were grown on coupons for 5 days, after which they were treated with either PBS (untreated control), Dual Zinc (0.96% zinc ions; zinc oxide and zinc citrate), or DZA (0.96% zinc ions and 1.5% l-arginine) for 2 min. Biofilm-coated coupons were then attached to the rheometer and spun at increasing velocities from 0.1 to 300 rad/s over 360 s and immersed in water. The resulting torque, a measurement of resistance to rotation of the coupon, was measured across the velocity range. This analysis revealed that treated S. gordonii biofilms displayed a reduced torque compared to untreated biofilms (Fig. 1A to C). This indicated that coupons coated with treated S. gordonii biofilms had less resistance to rotation compared to untreated biofilms. Furthermore, depicting these data as the mean of individual replicates revealed that the torque of S. gordonii biofilms treated with DZA was lower across the assayed velocity range compared to both the untreated and zinc-treated biofilms (Fig. 1D).

**FIG 2:**
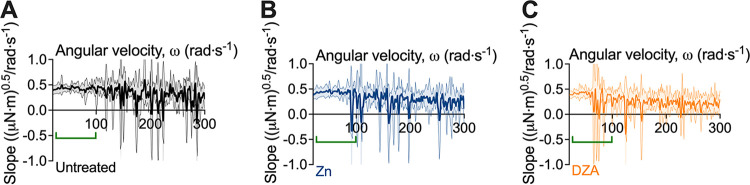
Transformed linearized analysis revealed early attachment events for Dual Zinc plus Arginine-treated S. gordonii biofilms. Transformed linearized analysis of data depicted in Fig. 1 for (A) untreated, (B) zinc (Zn)-treated, and (C) Dual Zinc plus Arginine (DZA)-treated S. gordonii biofilms. Data are presented as mean ± 95% confidence interval. Green brackets indicate regions where changes in torque, depicted here as negative slope values, were observed for DZA-treated biofilms but not for untreated or zinc-treated biofilms. Three biological replicates were performed each with duplicate biofilms.

We previously observed that reductions in torque during the test correlated with the detachment of biofilm aggregates from the coupon surface (17). These biofilm detachment events were similarly observed here (Fig. 1). To depict these events, a baseline correction was performed by which the data were linearly transformed and presented more clearly as the change in slope between measurements (i.e., an approximation of the first derivative), which enhanced the changes in torque as observed by the negative slope values (Fig. 2). This revealed that treatment of S. gordonii biofilms led to reductions in torque at lower velocity ranges compared to untreated biofilms (Fig. 2). Moreover, changes in torque at lower velocities were observed for biofilms treated with DZA, compared to untreated and zinc treated biofilms (Fig. 2; green bracket).

**FIG 3:**
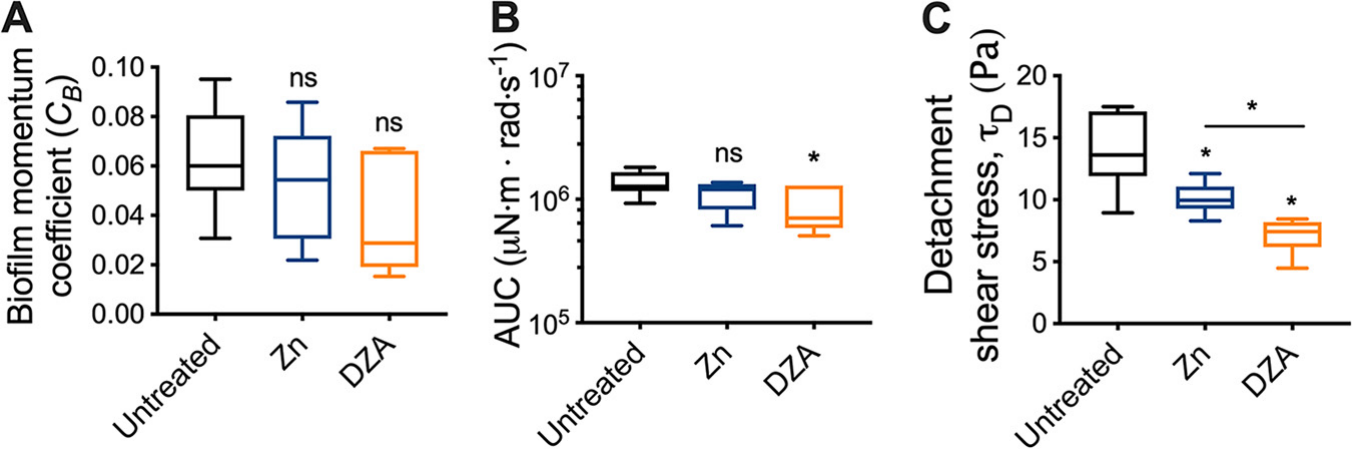
S. gordonii biofilms treated with Dual Zinc plus Arginine are more easily detached from surfaces. From the rotating-disc rheology analysis depicted in Fig. 1, the (A) biofilm momentum coefficient, (B) area under the curve (AUC), and (C) initiation of detachment shear stress that corresponded to the first decrease in torque were determined. Data are depicted as box and whisker plots of 3 biological replicates each with duplicate biofilms. *, *P* < 0.05; ns, not significant. Statistical comparisons are to the untreated control unless indicated in the graph.

To quantify these differences the biofilm momentum coefficient was determined, which is an indication of the biofilm-induced drag on rotation (17, 36, 37). Although there were no statistical differences between the biofilm momentum coefficient between treated and untreated biofilms, there was a general trend that both zinc- and DZA-treated S. gordonii biofilms had a reduced coefficient compared to untreated biofilms. This was more pronounced for DZA-treated biofilms (Fig. 3A; biofilm momentum coefficient mean ± SD; untreated 0.063 ± 0.021; Zn 0.053 ± 0.023; DZA 0.037 ± 0.023). This suggested that less drag was experienced by coupons coated with DZA-treated biofilms, which is associated with a less adhered biofilm (17, 36, 37). To investigate this further, the area under the curve (AUC) of the torque–angular velocity curves were measured, which is an indication of the amount of energy required for the coupon to rotate. This analysis revealed that only DZA-treated biofilms had a significantly reduced AUC compared to untreated biofilms (Fig. 3B), corroborating the trends observed from the biofilm momentum coefficient analysis (Fig. 3A). Together, these data indicated that coupons coated with DZA-treated biofilms experienced less drag compared to untreated and zinc treated biofilms, suggestive of a less adherent and mechanically weaker biofilm.

To determine if these observations correlated with differences in biofilm-aggregate detachment, the angular velocity where the first reduction in torque occurred was converted to a shear stress, indicative of the initiation or critical biofilm detachment shear stress (Fig. 3C). This revealed that reductions in torque, which correlated with biofilm-aggregate detachment, occurred at significantly lower shear stresses for treated S. gordonii biofilms compared to untreated biofilms (Fig. 3C). Furthermore, DZA-treated biofilms had significantly lower critical biofilm detachment shear stresses compared to zinc treated biofilms (Fig. 3C). This indicated that DZA-treated biofilms detached from the coupon surfaces at lower shear stresses compared to untreated and zinc treated biofilms, suggesting greater susceptibility to removal by external shear forces.

### Combined treatment with Dual Zinc plus Arginine destabilized the mechanical integrity of saliva-plaque biofilms.

Having demonstrated that treatment with DZA led to the mechanical destabilization of S. gordonii mono-species biofilms (Fig. 1 to 3), we wanted to determine the effects of DZA treatment using a biofilm model that more closely mimics *in vivo* conditions.

**FIG 4:**
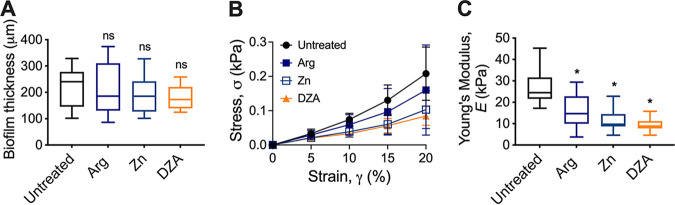
Treatment of *in vitro* saliva-plaque biofilms with Dual Zinc plus Arginine reduced the biofilm Young’s modulus. Saliva-plaque biofilms were grown for 5 days and treated with solutions of 1.5% arginine (Arg), 0.96% zinc (Zn), DZA, or PBS (untreated control) for 2 min. Treated biofilms were then analyzed using uniaxial mechanical indentation. (A) Biofilm thickness was determined from this analysis. Data are presented as a box and whisker plot. (B) The lower linear portion of stress-strain curves of saliva-plaque biofilms. Data presented as mean ± SD. (C) From this analysis, the Young’s modulus (stiffness) was also determined. Data are presented as a box and whisker plot. Four biological replicates each with three technical replicates. *, *P* < 0.05; ns, not significant. Statistical comparisons are to the untreated control.

To assess this, saliva-plaque biofilms were grown on hydroxyl-appetite (HA) discs for 5 days (38, 39). Biofilms were then treated with either solution of PBS (untreated control), 1.5% arginine, Dual Zinc, or DZA for 2 min. The biofilm mechanical properties were then assessed using uniaxial mechanical indentation, where biofilms were compressed at a defined rate and the resistance force measured. The biofilm thickness was determined by an increase of force in resistance to compression. The treatment did not change the biofilm thickness (Fig. 4A). The differences in the resulting stress-strain profiles revealed that all three treatments altered the biofilm mechanical properties relative to the untreated control (Fig. 4B). To therefore quantify the differences in the biofilm mechanical properties, the Young’s modulus, a measure of the ability of the biofilm to resist compression and a measure of biofilm stiffness (31, 40), was determined. This revealed that biofilms treated with either arginine, zinc, or DZA had a significantly lower Young’s modulus compared to the untreated control (Fig. 4C). This indicated that treatment with either solution destabilized the mechanical integrity of the saliva-plaque biofilm. While there was no statistically significant difference (*P* > 0.05) between either treatment, there was a trend where biofilms treated with arginine or zinc alone or DZA showed incremental decreases in the Young’s modulus. Biofilms treated with DZA displayed the lowest Young’s modulus (Young’s modulus mean ± SD; untreated 27.28 ± 8.68; arginine 15.38 ± 7.97; zinc 11.14 ± 5.02; DZA 9.29 ± 2.87). This suggested that treatment with DZA had the most impact on destabilizing the mechanical properties of saliva-plaque biofilms.

We were also interested in determining if the ability of DZA to destabilize the biofilm mechanical properties was maintained when developed into a dentifrice paste. Saliva-plaque biofilms were grown for 5 days and treated with either DZA paste, a commercially available stannous fluoride toothpaste, or PBS and analyzed by mechanical indentation as above. This revealed that biofilms treated with DZA dentifrice paste had a significantly lower Young’s modulus compared to untreated biofilm (Fig. S1 in Supplemental File 1), which was similar to what we observed for treatment with the test solutions (Fig. 4C). In contrast, biofilms treated with the stannous fluoride control paste had a significantly higher Young’s modulus compared to both untreated biofilms and biofilms treated with DZA (Fig. S1 in Supplemental File 1). This demonstrated that both the action of DZA on biofilms was maintained in a toothpaste formula and that the DZA technology may be more effective at reducing the mechanical integrity of saliva-plaque biofilms compared to other commercially available antibacterial dentifrices.

### Combined treatment with Dual Zinc plus Arginine degraded the EPS of saliva-plaque biofilms.

Biofilm mechanics is a property associated with the collective behavior of the biofilm that is largely imparted by the extracellular polymeric slime (EPS) (31). Having observed that treatment with either compound did not result in changes to the biofilm thickness (Fig. 4B), we therefore, wanted to determine if treatment with DZA exhibited an impact on biofilm EPS.

**FIG 5:**
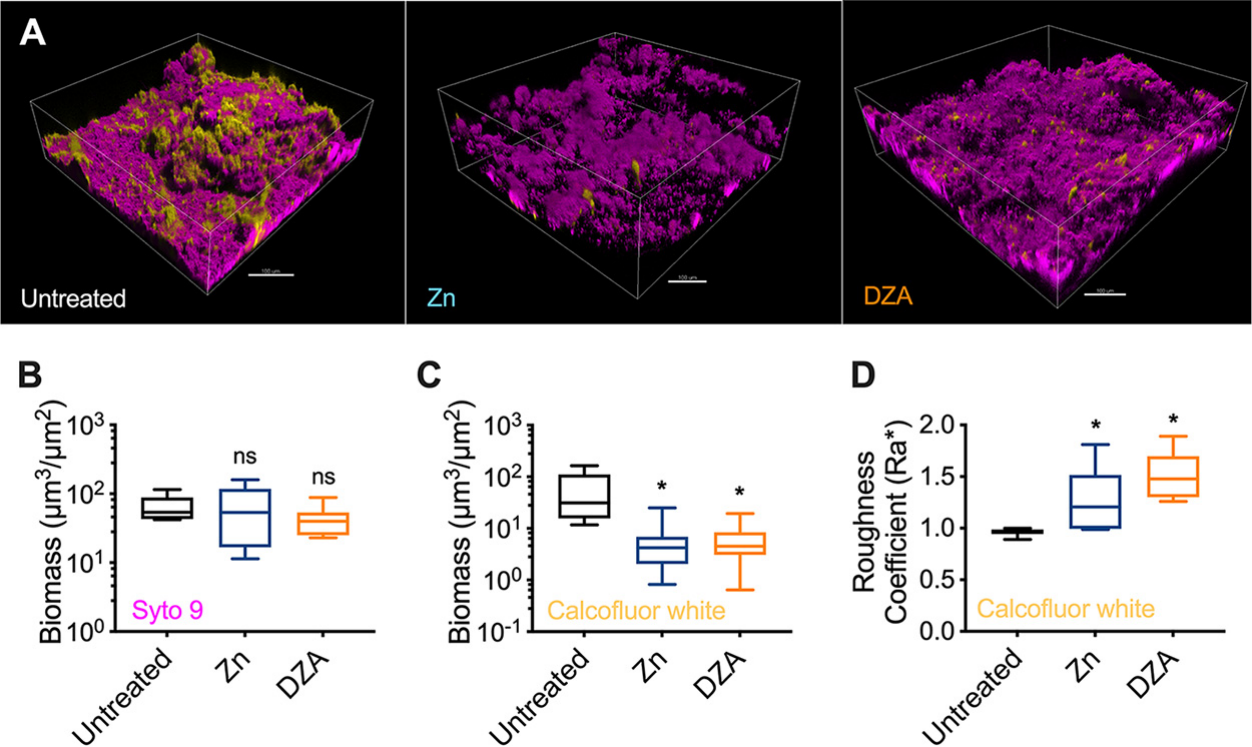
Treatment of saliva-plaque biofilms with Dual Zinc plus Arginine targeted the biofilm EPS. (A) Representative images of 5-day saliva-plaque biofilms either untreated (left) or treated with 0.96% zinc (Zn; middle) or DZA (right) for 2 min. Biofilms were stained with Syto 9 (magenta) and calcofluor white (yellow) to label the bacterial cells and the EPS, respectively. The scale bar indicates 100 μm. COMSTAT image analysis was performed to quantitate the (B) biomass of Syto 9 stained cells and the (C) biomass and (D) roughness coefficient of calcofluor white labeled EPS. Data presented as a box and whisker plot of 3 biological replicates, with 4 images per biofilm. *, *P* < 0.05; ns, not significant. Statistical comparisons are to the untreated control.

To assess this, 5-day saliva-plaque biofilms were treated with either PBS (untreated control), Dual Zinc, or DZA for 2 min. Treatment with arginine alone was omitted from this analysis because it showed an overall higher Young’s modulus compared to the other two treatments (Fig. 4C). Biofilms were then stained with Syto 9 to label the bacterial cells and calcofluor white to label the EPS and imaged using confocal laser scanning microscopy (CLSM). This revealed that treatment with either zinc or DZA appeared to reduce the amount of calcofluor white-labeled EPS within the biofilm (Fig. 5A).

To quantify these changes, both Syto 9 and calcofluor white staining was analyzed using COMSTAT (41, 42). Consistent with the biofilm thickness analysis from the uniaxial indentation measurements (Fig. 4B), neither treatment significantly altered the biomass of cells compared to the untreated control (Fig. 5B). However, calcofluor white-stained EPS revealed that treatment with either zinc or DZA showed a significant reduction in the volume of EPS within biofilms (Fig. 5C). Furthermore, the roughness coefficient, a measurement of the variability in height and heterogeneity (41, 42), of calcofluor white-labeled EPS of treated biofilms was significantly higher than untreated controls (Fig. 5D). This indicated that treatment with either zinc or DZA led to an overall reduction in calcofluor white-labeled EPS and changed the distribution of EPS from a more homogenous layer in untreated biofilms to a more heterogeneous punctate distribution in treated biofilms. Again, there was a trend that calcofluor white-labeled EPS of DZA-treated biofilms had a higher roughness coefficient compared to treatment with zinc alone (Fig. 5D). This suggested that DZA treatment led to a more uneven, heterogenous EPS distribution within the biofilm compared to treatment with zinc. However, this was not statistically significant.

## DISCUSSION

Treating biofilms with DZA is effective at reducing dental plaque biofilms *in vitro* and *in vivo* (5, 16, 29, 30). However, it is unclear how DZA impacts the structure and mechanical properties of dental plaque biofilms. Here, we demonstrated that DZA destabilized the mechanical integrity of both S. gordonii mono-species biofilms (Fig. 1 to 3) and dental plaque biofilms (Fig. 4 and Fig. S1 in Supplemental File 1) by targeting the EPS (Fig. 5). This correlated with biofilms being more susceptible to removal by shear forces (Fig. 1 to 3). Furthermore, we observed that these effects of DZA appeared to be increased compared to either zinc or arginine treatment alone. This suggests that there are synergistic or additive interactions between zinc and arginine that mediate the disruption of dental plaque biofilms.

**FIG 6:**
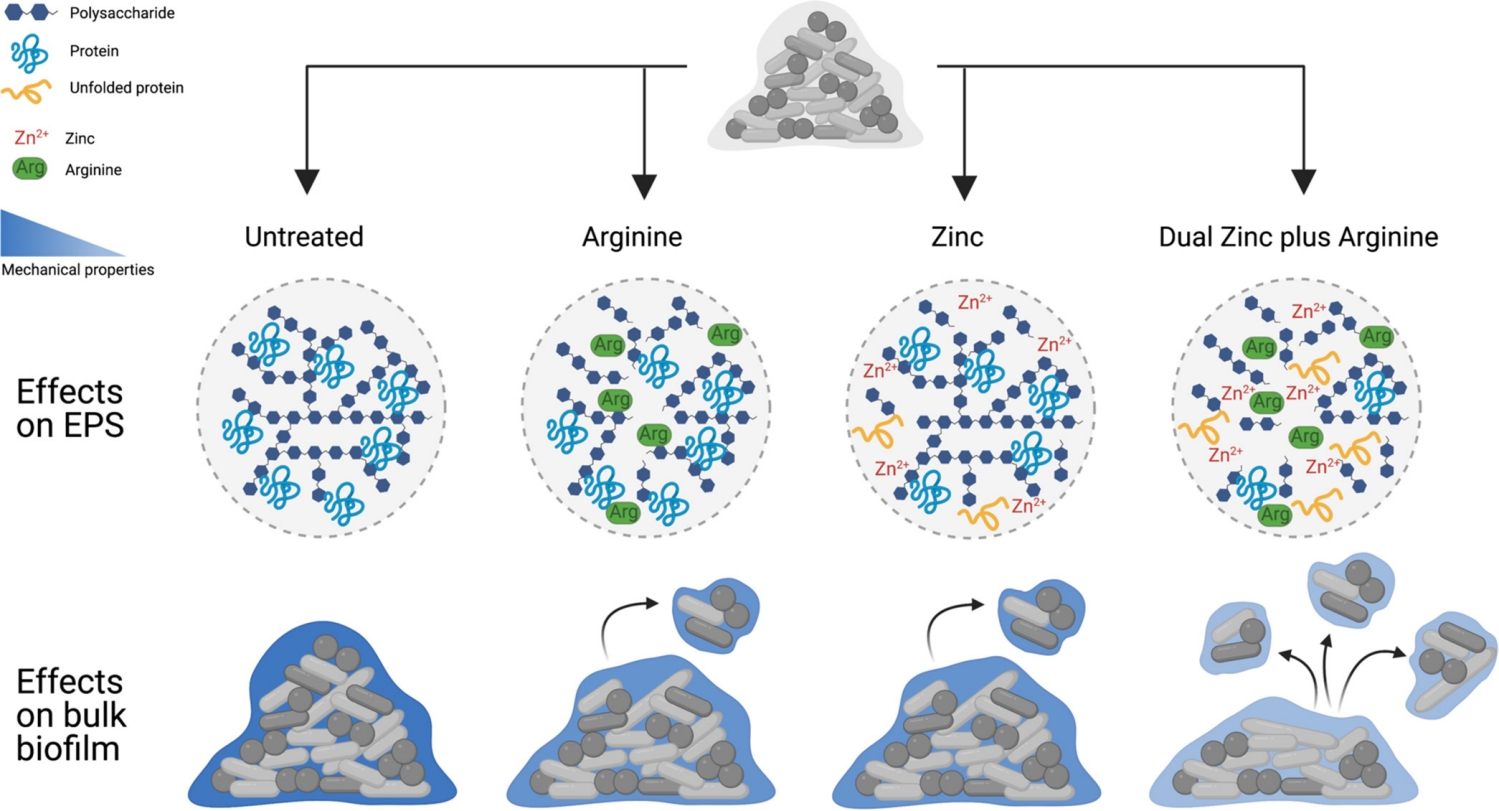
Proposed model for the effect of Dual Zinc plus Arginine treatment on biofilms. Dental-plaque biofilms are polymicrobial with an EPS comprising exopolysaccharides and proteins, lending wild-type mechanical properties to the biofilm (left). Treatment with either arginine (middle left) or zinc (middle right) alone destabilizes interactions between the EPS, leading to a comparable reduction in the biofilm mechanical properties between either treatment. When treated with Dual Zinc plus Arginine, arginine penetrates the biofilm, destabilizing the EPS, and allowing zinc to gain entry further into the biofilm. Subsequently, the actions of zinc are no longer localized to the biofilm periphery, amplifying the destabilization of the EPS, and reduction in the mechanical properties. These combined actions of DZA result in dental plaque-biofilms being more susceptible to removal by mechanical shear (right). Figure created in BioRender.com.

We propose the following model for potential interactions between arginine and zinc (Fig. 6). There is evidence to suggest that exogenous arginine can penetrate bacterial biofilms (43) and disrupt cell-cell and EPS interactions within the biofilm (10, 15). We, therefore, propose that arginine can penetrate the dental plaque biofilm, targeting interactions within the EPS and destabilizing the mechanical structure of the biofilm (Fig. 6). This is consistent with the reduced Young’s modulus observed for arginine-treated saliva-plaque biofilms compared to untreated biofilms (Fig. 4C). Furthermore, we previously reported that arginine-treated S. gordonii biofilms are more susceptible to removal by shear forces, due to the weakened mechanical structure (Fig. 6) (17). Here, we also demonstrated that treatment with zinc alone could weaken the mechanical structure of dental plaque biofilms (Fig. 4 and Fig. S1 in Supplemental File 1) by targeting the biofilm EPS (Fig. 5). Similarly, zinc has been shown to inhibit surface attachment and EPS production of Streptococcus mutans (44). However, the antimicrobial effects of zinc are reduced in biofilms compared to planktonic cultures, and it has been suggested that zinc is unable to penetrate the biofilm (19, 23, 26). We, therefore, propose that zinc can target the dental plaque biofilm EPS possibly through cationic interactions. However, this action is limited to the periphery of the biofilm (Fig. 6). We predict that zinc treatment weakens the mechanical structure of the biofilm to a similar extent as arginine treatment, which is consistent with the Young’s modulus of zinc- or arginine-treated saliva-plaque biofilms being similar (Fig. 4C). For treatment with DZA, we predict that arginine first penetrates and structurally weakens the biofilm through disruption of the biofilm EPS. Loosening the biofilm structure then permits zinc to gain entry deeper into the biofilm, extending the effects of zinc to deeper regions of the biofilm (Fig. 6). This results in further weakening of the biofilm mechanical structure and increased susceptibility of the biofilm to removal by shear forces, compared to treatment with either arginine or zinc alone. Supporting this model, matrix-assisted laser desorption/ionization-time of flight (MALDI-TOF) mass spectrometry (MS) analysis revealed that saliva-plaque biofilms treated with a DZA dentifrice had increased penetration and retention of zinc compared to treatment with Dual Zinc alone (16). This model currently only takes into consideration the physical effects over the short time scales (2 min) analyzed here.

To determine if the mechanical destabilization of dental plaque biofilms was due to synergistic or additive interactions between zinc and arginine, we applied a modified fractional inhibitory concentration (FIC) index, referred to here as the interaction index. The FIC index is traditionally used to determine whether the interactions between antimicrobials are synergistic, additive, or antagonistic when used in combination (45, 46). By substituting the MICs for the Young’s modulus, according to equations 2 to 4, we obtained an interaction index of 1.87 ± 0.95 (mean ± SD) for DZA, suggestive of additive interactions (45, 46). Similarly, when the Young’s modulus of arginine-treated and zinc-treated saliva-plaque biofilms are subtracted, we obtained a value (4.95 ± 3.30) more similar to the Young’s modulus of DZA-treated biofilms (9.29 ± 2.87) compared to when moduli were divided (1.51 ± 0.35). This further supported that the interactions between zinc and arginine were additive. This suggests that across the short time scales analyzed here (2 min), both arginine and zinc have a similar target, presumably EPS (Fig. 5), that is responsible for the mechanical destabilization of the biofilm. However, across longer time scales with repeat treatments, it is likely that the antimicrobial effects of zinc and the metabolic effects of arginine will influence these interactions, potentially shifting them to being more synergic. Therefore, future studies that focus on these interactions, across longer time scales, are warranted. Furthermore, this study focused on the maintenance of oral health hygiene, rather than the removal of pathogenic oral biofilms. Therefore, biofilms formed by oral commensals (S. gordonii) and saliva-plaque biofilms grown from healthy donors were only examined here. Future studies that focus on the cariogenic oral biofilm pathogen S. mutans grown in the presence of sucrose are also warranted to determine if these interactions between zinc and arginine are maintained in the treatment of a cariogenic biofilm, which include a sticky glucan EPS matrix and acidic environment.

Importantly, long-term (6 months) clinical trials of a dentifrice containing DZA did not report any adverse side effects among the 100 participants (30). Similarly, long-term studies (>1 year) of zinc-supplemented dentifrices reported no significant changes in oral microbial ecology or antimicrobial-resistant bacteria (47, 48). Our results and proposed model suggest that targeting dental plaque biofilm mechanics is a strategy to enhance the action of actives against bacteria within dentifrices. Our results are also supported by similar observations, where arginine increased the susceptibility of saliva-plaque biofilms to cetylpyridinium chloride (15), Streptococcus pyogenes biofilms to a penicillin (49), and Pseudomonas aeruginosa biofilms to tobramycin and ciprofloxacin (43). Our results, therefore, support the growing evidence that arginine has the potential to be used as an antimicrobial adjuvant. Finally, zinc oxide inhibits the biofilm formation of P. aeruginosa, Chromobacterium violaceum (50), and Candida albicans (51). This suggests that DZA has the potential to be used across diverse clinical applications for the treatment of biofilm-associated infections, in addition to the maintenance of oral health and hygiene.

## MATERIALS AND METHODS

### Preparation of treatment formulations.

Test solutions consisted of 1.5% arginine, Dual Zinc (0.96% total zinc ions; zinc citrate and zinc oxide), or Dual Zinc plus Arginine (0.96% zinc ions; zinc oxide and zinc citrate, and 1.5% l-arginine). Solutions were adjusted to a final pH of 7.0. In toothpaste-treated biofilm experiments, the biofilms were treated with either commercially available stannous fluoride 0.0454% based active agent stabilized with sodium hexametaphosphate (Procter and Gamble, UK) or Colgate Total containing Dual Zinc plus Arginine and (Colgate-Palmolive, UK).

### Saliva-plaque inoculum collection.

Saliva plaque was collected from healthy individuals according to protocols approved by the Ohio State University (OSU) Institutional Review Board (IRB) (number 2017H0016, 03/22/2017) (39). Collected samples were pooled and separated into 1 mL aliquots supplemented with 30% glycerol. Samples were stored at −80°C before experimentation.

### Biofilm growth.

**(i) S. gordonii biofilms.** S. gordonii wild-type strain DL1 was used in this study. Biofilms were grown as previously described (17). Briefly, sterile 40 mm diameter coupons were submerged in 40 mL brain heart infusion media, supplemented with 0.5% sucrose, and inoculated with 400 μL of overnight S. gordonii culture. Coupons were incubated in a humidified chamber at 37°C with 5% CO_2_ and on an orbital shaker at 150 rpm. The growth medium was replaced every 24h. Biofilms were grown for 5 days.

**(ii) *In vitro* saliva-plaque biofilms.** Sterile hydroxyl-appetite (HA) discs were transferred to a 24-well plate, one disc per well. Next, 900 μL of McBain media (2g/L peptone, 2g/L Trypticase peptone, 1g/L yeast extract, 0.35g/L NaCl, 0.2g/L KCl, 0.2g/L CaCl_2_, 2.5g/L mucin, 50 mM PIPES buffer, 5 μg/mL hemin, and 1 μg/mL vitamin K) was added to each well and inoculated will 100 μL of pooled saliva-plaque. The plate was incubated in a humidified chamber at 37°C with 5% CO_2_ on an orbital shaker at 150 rpm. Every 24h the growth medium was replaced with 1 mL of fresh McBain media. Biofilms were grown for 5 days. This model is supportive of the growth of early colonizer aerobes and late colonizer anaerobes within the saliva-plaque biofilm (38, 39).

### Biofilm treatment.

**(i) Treatment with solutions.** After 5 days of growth, S. gordonii biofilm-coated coupons were transferred to a dish containing 60 mL of either PBS (untreated control), Dual Zinc (0.96% zinc ions), or DZA. Biofilms were treated for 2 min at 37°C with 5% CO_2_ and shaking at 150 rpm. Biofilms were then washed twice in PBS before being transferred into 60 mL PBS until analysis.

Saliva-plaque biofilm-coated hydroxyl-appetite (HA) discs were treated as above except biofilms were transferred to a new 24-well plate, with each well containing 1 mL of either PBS (untreated control), 1.5% arginine, Dual Zinc, or DZA. After treatment biofilms were washed twice in PBS, before being transferred into 1 mL PBS until analysis.

**(ii) Treatment with pastes.** One gram of DZA paste was dissolved in 4 mL sterile water (1:5 vol/vol dilution). One gram of commercially available stannous fluoride toothpaste was also dissolved in 4 mL of sterile water and used as a control paste treatment. The foam was allowed to settle before 1 mL of paste suspension was transferred to wells of 24-well plate. HA discs coated with 5-day saliva-plaque biofilms were transferred into wells containing paste suspension or sterile water (untreated control). Biofilms were treated for 2 min at 37°C with 5% CO_2_ and shaking at 150 rpm. Biofilms were then washed twice in PBS, before being transferred into 1 mL PBS until analysis.

### Rheometry analysis.

**(i) Adapted rotating-disc rheology.** S. gordonii biofilms were analyzed using an adapted rotating-disc rheology assay as previously described (17). Briefly, biofilm-coated coupons were attached to the shaft of a Discovery Hybrid Rheometer-2 (DHR-2) (TA Instruments). Biofilms were immersed in a 15 × 15 cm square clear acrylic container filled with 2.8 L reverse osmosis water with the gap distance set to 3.5 cm. Immersed coupons were spun at an angular velocity (ω) range of 0.1 to 300 rad/s, incrementing the speed across 360 s. For each biofilm treatment (untreated, Dual Zinc, or DZA), 3 biological replicates were performed, each with duplicate biofilms (a total of 6 biofilms analyzed per treatment group).

The torque–angular velocity curves were linearized and transformed to more clearly depict the data as previously described (17). The fouling coefficient and conversion of torque to shear stress were calculated as previously described (17). The area under the curve (AUC) of the torque–angular velocity curves were determined using the analysis function within GraphPad Prism v8.

**(ii) Uniaxial mechanical indentation.** Biofilms were analyzed on a DHR-2 with a Peltier plate connected to a heat exchanger (TA Instruments). Biofilms were analyzed and submerged in PBS. Indentation measurements were performed at 25°C using the 8 mm Smart Swap geometry with an approach rate of 1 μm/s and a termination detection at 15 N. Contact with the biofilm was determined where the force began to steadily increase. Young’s modulus (E) was calculated using the force-displacement relationship previously described (40).

(1) $$E=\frac{\mathrm{slope}(1-v^{2})}{2r}$$

The slope is of the force-displacement curve (N/m), *r* is the radius of the probe (*r *= 0.004m), and *v* is the assumed Poisson’s ratio of a biofilm (*v *= 0.5) (52). The slope of the force-displacement curve was measured at the region corresponding to 0 to 20% strain. Four biological replicates, each with three technical replicates, were performed for each treatment for both solutions and paste suspensions (a total of 12 biofilms analyzed for each treatment group).

To determine if the combined actions of DZA to the biofilm mechanical properties were synergistic or additive, we used the fractional inhibitory concentration (FIC) index, referred to here as the interaction index (IAI), which has traditionally been used to determine the interactions between multiple antimicrobials (45, 46). However, we substituted the MIC for the Young’s modulus, according to equation 2 to 4.

(2) $$\mathrm{IA}I_{\mathrm{Arg}}=\frac{E_{\mathrm{DZA}}}{E_{\mathrm{Arg}}}\;$$

(3) $$\mathrm{IA}I_{\mathrm{Zn}}=\frac{E_{\mathrm{DZA}}}{E_{\mathrm{Zn}}}$$

(4) $$\mathrm{IAI}=\mathrm{IA}I_{\mathrm{Arg}}+\mathrm{IA}I_{\mathrm{Zn}}$$

Here, IAI ≤ 0.5 was indicative of synergism, IAI > 0.5 to 4 was indicative of additive, and IAI ≥ 4 was indicative of antagonism.

### Confocal laser scanning microscopy.

For confocal laser scanning microscopy (CLSM), treated 5-day saliva-plaque biofilms were transferred to 1 mL 4% paraformaldehyde and fixed at room temperature for 1 h. Biofilms were then transferred to 1 mL PBS supplemented with 5 μM Syto-9 and 10 μg/mL calcofluor white and incubated for 1 h at room temperature. Biofilms were washed twice in PBS and transferred to fresh PBS until imaging. Biofilms were imaged on an Olympus FV1000 Multiphoton microscope, in single photon mode, fitted with a 25× water immersion objective. Images were processed using the Imaris software and analyzed using COMSTAT-2 (41, 42). For each biofilm treatment (untreated, Dual Zinc, or DZA), 3 biological replicates were imaged, with 4 images taken per biofilm.

### Statistical analysis.

Statistical analysis was performed using a one-way analysis of variance ANOVA with Tukey’s *post hoc* test. Analyses were performed using GraphPad Prism v8 (GraphPad Software). Statistical significance was determined using *P* < 0.05.
