# Supplementary material for: A Combination of Zinc and Arginine Disrupt the Mechanical Integrity of Dental Biofilms
Source: Microbiol Spectr. 2022 Dec 6;11(1):e03351-22. doi: 10.1128/spectrum.03351-22 (PMC9927089; doi:10.1128/spectrum.03351-22)
Supplement: Supplemental file 1 — Figure S1. Download spectrum.03351-22-s0001.pdf, PDF file, 0.1 MB [file spectrum.03351-22-s0001.pdf]

A combination of zinc and arginine disrupt the mechanical integrity of dental biofilms

Supplemental Information

Erin S. Gloag, Yalda Khosravi, James G. Masters, Daniel J. Wozniak, Carlo Amorin Daep, Paul Stoodley

Supplemental Figures

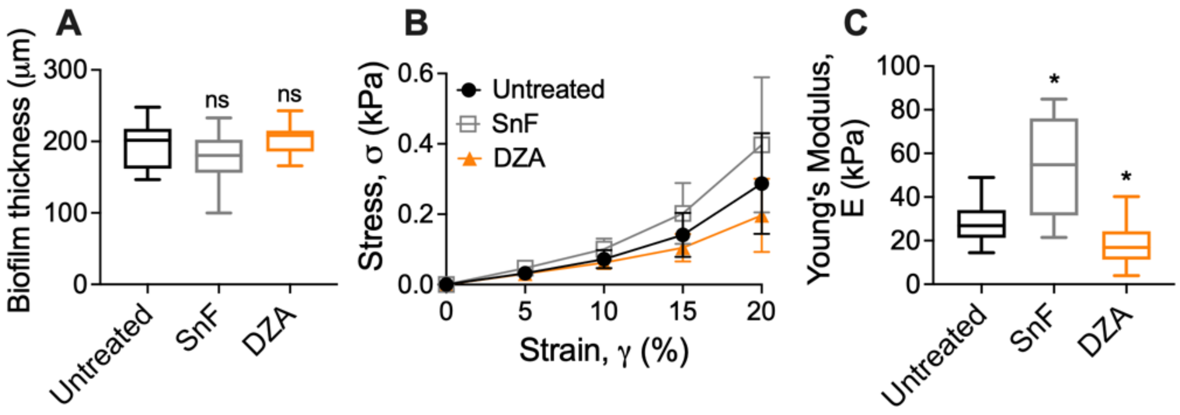

Supplemental Figure 1: Comparison of Dual Zinc plus Arginine dentifrice to a control stannous fluoride dentifrice. Saliva-plaque biofilms were grown for 5 days and treated with either Dual Zinc plus Arginine (DZA) dentifrice paste or a commercially available stannous fluoride toothpaste (SnF), or sterile water (untreated) for 2 min. Biofilms were then analyzed using uniaxial mechanical indentation. **(A)** Biofilm thickness was determined from this analysis. Data presented as a box and whisker plot. **(B)** Lower linear portion of stress-strain curves of saliva-plaque biofilms. Data presented as mean ± SD. **(C)** From this analysis the Young's modulus was also determined. Data presented as a box and whisker plot, of 4 biological replicates, each with 3 technical replicates. * p-value <0.05; ns indicates not significant. Statistical comparisons are to the untreated control.
